# What are the living conditions and health status of those who don’t report their migration status? a population-based study in Chile

**DOI:** 10.1186/1471-2458-12-1013

**Published:** 2012-11-21

**Authors:** Baltica Cabieses, Kate E Pickett, Helena Tunstall

**Affiliations:** 1 Lecturer at the Faculty of Medicine, Universidad del Desarrollo, Av. Las Condes 12.438, Lo Barnechea, Santiago, Chile; 2 Visiting scholar at the Department of Health Sciences, University of York, Second Floor ARRC Building, Heslington, York, YO10 5DD, UK; 3 Professor at the Department of Health Sciences, University of York, Seebohm Rowntree Building, Area 3 Heslington, York, YO10 5DD, UK; 4 Research Fellow at the School of GeoSciences, University of Edinburgh, Geography Building, Drummond Street, Edinburgh, EH8 9XP, UK

**Keywords:** Self-reported health, Population-based study, Missing data, Immigrants, Undocumented immigration, Socioeconomic status

## Abstract

**Background:**

Undocumented immigrants are likely to be missing from population databases, making it impossible to identify an accurate sampling frame in migration research. No population-based data has been collected in Chile regarding the living conditions and health status of undocumented immigrants. However, the CASEN survey (Caracterizacion Socio- Economica Nacional) asked about migration status in Chile for the first time in 2006 and provides an opportunity to set the base for future analysis of available migration data. We explored the living conditions and health of self-reported immigrants and respondents who preferred not to report their migration status in this survey.

**Methods:**

Cross-sectional secondary analysis of CASEN survey in Chile in 2006. O*utcomes*: any disability, illness/accident, hospitalization/surgery, cancer/chronic condition (all binary variables); and the number of medical/emergency attentions received (count variables). *Covariates*: Demographics (age, sex, marital status, urban/rural, ethnicity), socioeconomic status (education level, employment status and household income), and material standard of living (overcrowding, sanitation, housing quality). Weighted regression models were estimated for each health outcome, crude and adjusted by sets of covariates, in STATA 10.0.

**Results:**

About 1% of the total sample reported being immigrants and 0.7% preferred not to report their migration status (Migration Status - Missing Values; MS-MV). The MS-MV lived in more deprived conditions and reported a higher rate of health problems than immigrants. Some gender differences were observed by health status among immigrants and the MS-MV but they were not statistically significant. Regressions indicated that age, sex, SES and material factors consistently affected MS-MVs’ chance of presenting poor health and these patterns were different to those found among immigrants. Great heterogeneity in both the MS-MV and the immigrants, as indicated by wide confidence intervals, prevented the identification of other significantly associated covariates.

**Conclusion:**

This is the first study to look at the living conditions and health of those that preferred not to respond their migration status in Chile. Respondents that do not report their migration status are vulnerable to poor health and may represent undocumented immigrants. Surveys that fail to identify these people are likely to misrepresent the experiences of immigrants and further quantitative and qualitative research is urgently required.

## Background

Migration is a complex global phenomenon with incompletely understood health effects [1]. Research addressing the health of immigrants has been hampered by the quality of available data. Several national-level studies in Latin America, Asia, Spain, the US and Australia have found a significant under-representation of immigrants in comparison to government estimations [2-4]. This under-representation frequently relates to undocumented immigrants, a group that are likely to be missing from population databases, making it impossible to identify a complete, accurate sampling frame for use in migration research. Under-representation is also particularly significant for some specific hard to reach groups like vulnerable women, children and minority ethnic people [5,6]. Some limited evidence indicates that these undocumented immigrants tend to live in higher socioeconomic deprivation, social isolation, and poorer health conditions than the local and the documented immigrant populations [7,8]. They also report lack of access and limited use of health care [9]. Relatively little is known however about undocumented migrants worldwide [10,11], including those in the Latin American region [12-14]. Further research regarding migrants is urgently required to improve the living conditions and health of these populations, and especially those living in socioeconomic vulnerability, such as undocumented immigrants.

Chile has been receiving an increasing number of international immigrants in recent years, representing approximately 2% of the total population in the country in 2007 [15]. Chile’s growing appeal to international migrants reflects its status as an economically expanding middle-income country with an intermediate level of development and a stable economy [16]. This development has been accompanied by a progressive improvement in the health status of its population, with a decline in the infant and general mortality rates, and an increase in life expectancy [17-19]. Changes in the global economy and Chile`s social stability and steady economic growth could lead in coming years to a further increase in its immigration rates.

Current research on the living conditions and health of the immigrant population in Chile is scarce. National surveys, for example, have only recently started to include questions on migration status [20]. However, the limited evidence available identifies some key characteristics of migrants. Data for 2006 suggests immigrants compared to the Chilean-born have distinctive patterns of highly polarised socioeconomic status (SES) [21]. On average, immigrants earn three times more than the Chilean-born, but the bottom income quintile earns less than the equivalent Chilean-born poorest quintile. Immigrants have a significantly wider gap therefore between the wealthiest and the poorest income groups than the native population (a 23-fold gap versus 13-fold gap, respectively, 20% top: 20% bottom ratio). Regarding types of occupation, employed immigrants have a 1.7 times higher proportion of people with managerial/executive occupations than employed Chilean-born. However, immigrants also report a 2.9 fold higher rate of people in domestic service and a lower proportion of private employee occupations than the Chilean-born [21].

Other key features of international immigration to Chile, as reported by governmental figures from 2007, are: (i) immigrants are mostly Andean, with over 50% of them coming from Peru, Argentina and Ecuador; (ii) increasing labour force immigration, with economically active immigration growing from 31% in 1992 to 48% in 2002; (iii) increasing non-professional immigration, in line with the emerging world trend, with a reduction of professional and technical immigrants from 64% in 1992 to 45% in 2002; (iv) greater female immigration to work in unskilled/semiskilled manual occupations and domestic services [15,22]. In addition, evidence from qualitative studies suggests that a significant proportion of immigrants, including undocumented immigrants, do not have any health insurance and live in socioeconomic deprivation [12,23,24].

Three possible sources of quantitative data exist to study migrants in Chile; these being information from the Migration Department in the Chilean Government, Census data, and, since 2006, the CASEN survey (Caracterizacion Socio-Economica Nacional). The first has significant limitations for the measurement and exploration of undocumented migration and the second, the Census, while one of the best sources of data on demographic characteristics, does not provide a wide range of information on health status to relate to the socioeconomic determinants of the migrant population. The CASEN survey contains detailed health and socio-demographic data but also presents significant limitations, in particular, it has a high rate of missing values on migration status (MS-MV) and does not contain data on undocumented immigrants [25]. We selected the 2006 CASEN survey for analysis as this was the first survey in Chile to include a question on migration status and allows us to set the basis for further analysis on the living conditions and health status of international immigrants and people who don’t report their migration status. Recent studies have explored the living conditions and health of those who reported being international immigrants in the CASEN survey and compared them to the non-immigrant Chilean population [21,26,27], but no study has focused on those who did not report their migration status.

The question of whether the MS-MV correspond to international immigrants, potentially undocumented ones, cannot be answered directly through this analysis. There is no information in the CASEN survey data or administrative records to indicate whether the migration question missing values respondents or those who did report being international immigrants are undocumented immigrants. Nonetheless, international evidence has suggested that those that prefer not to report their migration status are commonly undocumented immigrants in fear of future prosecution [11,28,29] and under-estimation of immigrants in national representative surveys has been frequently reported in the past [1,30,31]. This group has not been explored in Chile previously and, therefore, this study focuses on the demographic, socioeconomic, material living conditions, and health status of those who preferred not to report their migration status; and how these patterns compare to those who reported being international immigrants. It aims to assess whether people that do not report their migrant status are a distinctive group and therefore if their omission from the data may obscure the complex characteristics of migrant populations.

## Methods

### Population and sample

Secondary data analysis of a nationally representative survey conducted in Chile in 2006 (CASEN). The CASEN survey was carried out every two years between 1987 and 2000, and subsequently has been conducted every three years [20,32]. It employed multistage probabilistic sampling with two phases (county and household), stratified by urban and rural area. Further detail of the sampling strategy and data collection has been presented elsewhere [20]. The final sample for the analysis consisted of 268 873 people who belonged to a random sample of 73 720 households, representing a 95.4% of the total Chilean territory [33]. The probabilistic sample had a final absolute sample error of 0.36% at the household level, assuming a confidence level of 95% and maximum variance [20]. The mean number of households included in the CASEN per region was representative of the total population within each region and also representative of the population in each urban and rural setting from each region [34]. The response rate of the 2006 CASEN survey was 84.8% [20].

### Migration status and the missing data

The CASEN 2006 survey asked: in which country was your mother living when you were born? Those who answered “in a different country from Chile” were identified as self-reported international immigrants, regardless of the country of origin of their parents (including Chilean people giving birth abroad). Approximately 0.9% of the total sample (n = 1877) was identified as self-reported immigrants, in contrast to the 1.8% estimated immigrant population according to Chilean government national statistics (National Institute of Statistics in Chile, 2008) [15,35]. An additional 0.7% preferred not to report their migration status (n = 1477). The self-reported immigrants and the missing values from the migration status question (Migration Status-Missing Values: MS-MV) are the two comparison groups in this study.

The CASEN survey has very high response rates for virtually all questions with an average rate of missing data below 0.05%, which was marginal for statistical analysis and therefore no multiple imputation technique was used. The question regarding migration status was, however, distinctive, as it had the greatest number of missing values of any of the questions in the survey (0.7%).

### Self-reported health outcomes

a.  Disability in the past year (yes/no): indicating the presence of one or more disabling conditions (visual, hearing, speaking, physical, cognitive, and psychiatric disability).

b.  Health problem or accident in the past month (yes/no): the presence of any health problem or accident in the past month.

c.  Hospitalization or surgery in the past year (yes/no): any hospitalization or surgery in the past year.

d.  Chronic condition or cancer in the past year (yes/no): the presence of these health events during the last 12 months.

e.  Number of medical attentions received in the past month (count variable, range 0–25): over-dispersed variable (equidispersion test p-value < 0.05) with a large number of zero values [36].

f. Number of emergency attentions received in the past month (count variable, range 0–28): over-dispersed variable (equidispersion test p-value < 0.05) with a large number of zero values [36].

### Demographic factors

These include age (continuous variable and categorical by three age-groups: under 15/ 15 to 64/ over 64), sex (binary variable, male/female), marital status (multinomial variables with four categories: single, married/cohabitant, separated/divorced, widow), urban versus rural area (binary variable), area of the country (multinomial with three categories: northern, central and southern), and belonging to any of the nine legally recognised pre-Hispanic minority ethnic groups in Chile (yes/no).

### Socioeconomic status (SES)

a. Household income: obtained from the total household income per capita in the past month in Chilean pesos and converted to USD purchasing power parity for 2006 (PPP, continuous variable, USD$1 corresponds to 531 Chilean pesos in 2006 currency) [16].

b. Educational level: collected by CASEN survey as the highest level for each adult (18 years old or older) member of the household (categorical variable): university, technical, high school, primary school or no education. Young adults aged between 18–22 years old with uncompleted higher education were included in the overall “university level” category.

c. Contractual status (yes/no): indicating whether the head of the household reports having a contract at the time of the interview.

### Material living standards

Household material living conditions were measured using three self-reported variables recommended by the Chilean Ministry of Planning [20] and the Households Assets Index:

a. Housing quality: index that combined quality of the walls, floor and ceiling. Housing quality was then categorised as high (constructed of solid materials), regular (poor quality but all enduring materials) and poor (constructed of one or more non-enduring materials, such as plastic or cardboard, which is frequently used by those living in transient camps in Chile).

b. Sanitary systems: measured by the presence of a clean public water supply and a public sewage system. A sanitary system was deficient when one or both of these measures were absent, irrespective of urban/rural location. An adequate sanitary system was defined as 0, and a deficient sanitary system was labelled as 1.

c. Overcrowding: binary variable defined using the Townsend measure of deprivation [39], which considers the ratio of the number of total rooms in the household over the total number of household members (a value of 1 indicates a ratio above 1).

d. Household assets index (HAI): continuous variable obtained from the combination of nine household assets through principal component analysis (PCA), which accounted for 48% of the total variance [37,38]. This index ranged between −1.00 and 9.87. The nine assets were: car, washing machine, fridge, water heater, land phone, cable TV connection, computer, internet access, and mobile phone.

### Analysis

Descriptive statistics for each self-reported health outcome under study were reported as means for continuous variables and proportions for categorical variables. Rates of each health outcome, crude and stratified by age groups and sex, were also reported, with corresponding Chi2 and t-tests for independent samples.

We aimed to understand the differences in socio-demographic characteristics between immigrants and the MS-MV and also how their health patterns differ. For this reason, we conducted separate regression analysis among immigrants and the missing values group, and then compared patterns between these subgroups. This allowed us to observe detailed variations in factors associated with health outcomes between the comparison groups. That is, this kind of analysis was appropriate as we were interested in effect modification of the associations between demographic, SES, or household material standards, and health outcomes.

Crude and adjusted Odds Ratios (OR, logistic regressions in the case of binary variables) and Prevalence Rate Ratios (PRR, zero-inflated negative binomial regressions in the case of count variables), with their 95% confidence intervals (robust standard errors method applied), were estimated by regression models. These models estimated the crude probability of presenting each health outcome of interest in the immigrant and the MS-MV groups. Given the large number of possible covariates to include in a single model and their multi-collinearity, we decided to conduct separate models by sets of factors (demographics, SES, and material living standards) crude and adjusted by sex, age and zone. This was also supported by the lack of previous information regarding the living conditions and factors associated with poor health in these two groups of interest, and therefore this exploratory approach to analysis was intended to raise new hypothesis for future testing in Chile and the region. We did not perform further statistical analysis in order to prevent the issue of multiple testing and the increased risk of spurious associations [40,41].

With regard to post-estimation tests of the regression models, we tested overall statistical association between covariates with multiple categories and health outcomes through the adjusted Wald test (a p-value <0.05 represented a significant association between the overall variable and the health outcome of interest) [42,43]. The Archer and Lemeshow goodness of fit test for a logistic regression model fitted using survey sample data was estimated (F-adjusted mean residual GOF test) [44]. A GOF test p-value above 0.05 (non significant) suggested a good fit of the model. For the zero-inflated negative binomial regressions, the test of the over-dispersion parameter alpha was estimated. When the over-dispersion parameter is zero the negative binomial distribution is equivalent to a Poisson distribution and when alpha is significantly different from zero (p value <0.05) it supports a good fit of the model over the Poisson regression [45]. In addition, the Vuong statistic test was used to assess whether a zero-inflated regression had a better fit than a non zero-inflated regression (p value <0.05 indicates a good fit of the zero-inflated model) [45].

Additionally, Exploratory Factor Analysis (EFA) was conducted in order to assess the correlation between the different health outcomes and their potential integration in a single score in the MS-MV group. Results showed that their correlation was poor (below 0.30) and all factor loadings were highly unique (above 0.80) indicating it would be better for the health outcomes to be explored separately [46]. Data analyses were conducted with the STATA 10.0 statistical software package and estimations were weighted with the use of “svy” commands to take into account the complex multistage sampling strategy of the survey and to attain population-based estimations [47].

### Ethics approval

This study obtained ethical approval from the University of York Research Governance Committee in 2009.

## Results

### Crude comparison of the demographics and living conditions of the MS-MV and the immigrant population

People that did not respond to the immigration status question (MS-MV) compared to self-reported immigrants had a younger mean age (26 versus 33 years-old) and were more likely to be children (45% versus 13%), especially among those that belonged to an ethnic minority group (40% versus 11%, data not shown). They were more likely than immigrants to live in rural areas (9% versus 6%) and in the Southern area of the country (20% versus 13%). The MS-MV group has a 9.2 times higher proportion of adult people with no education (21% versus 2%) and a 2.8 times higher proportion of people with education up to primary level only, compared to the immigrant group (33% versus 18%). Immigrants report a 2.2 times higher mean household income per capita per month (USD$746 versus USD$329) and a higher mean number of household assets than the MS-MV group (comparing the HAI between groups). A higher proportion of people in the MS-MV group live in overcrowded housing compared to immigrants (36% versus 25%) (Table 1).

**Table 1:** Living conditions of the MS-MV and the immigrant population (CASEN survey 2006)

	**Immigrant population 1% total sample**, **n = 154 431 weighted population(1877 real observations)**		**MS**-**MV GROUP 0**.**67% total sample**, **n = 108 599 weighted population(1477 real observations)**	
	**% or mean**	**95% CI**	**% or mean**	**95% CI**
*DEMOGRAPHICS*				
Mean age**	*X* = 33.41	31.81–35.00	X = 26.13	23.41–28.26
Age categories:				
<16 years old**	13.60	11.29–16.28	45.25	39.53–51.10
16-65 years old**	79.08	75.92–81.93	47.26	41.64–52.94
>65 years old	7.32	5.33–9.97	7.49	5.31–10.46
Sex (female = 1)	45.21	41.74–48.72	51.27	47.99–55.41
Marital status:				
Single**	45.81	42.06–49.62	64.30	59.36–68.95
Married**	45.49	41.66–49.36	29.39	25.09–34.10
Divorced*	4.21	3.06–5.77	2.23	1.32–3.74
Widow	4.49	2.89–6.91	4.07	2.55–6.44
Ethnicity: any	5.57	3.79–8.10	5.59	3.90–7.96
Zone (Rural = 1)**	6.03	4.89–7.42	9.99	7.87–12.59
Area:				
Northern	13.15	10.14–16.89	21.51	14.97–29.91
Central**	73.66	69.22–77.66	57.70	50.31–64.77
Southern*	13.19	10.50–16.45	20.78	16.41–25.96
*SOCIOECONOMIC STATUS*				
Educational level:				
No education**	2.38	1.51–3.73	21.96	18.64–25.68
Primary School**	18.79	16.05–21.88	33.92	29.58–38.55
High School**	33.02	29.39–36.87	18.80	15.34–22.83
Technical level**	16.81	14.13–19.88	8.86	6.59–11.80
University level**	27.32	23.16–31.98	7.98	4.75–13.11
Household income per capita (USD)**	X = 746.69	610.98–882.41	X = 329.05	265.92–392.02
Current worker (yes)**	60.96	57.06-64.73	71.96	67.28–76.21
*MATERIAL HOUSEHOLD CONDITIONS*				
Quality Household:				
Acceptable	75.59	71.21–79.51	76.33	70.85–81.03
Sub-standard	23.03	19.18–27.40	23.19	18.52–28.63
Unfit	1.37	0.83–2.27	0.48	0.28–0.81
Sanitary Index (adequate = 1)	9.33	7.34–11.80	13.02	10.40–16.19
Overcrowding**	25.79	21.51–30.58	36.96	30.65–43.75
HAI	*X* = 1.05	0.79–1.31	X = −0.11	−0.32 – -0.09

*p value <0.05 when comparing the same category between MS-MV and the immigrant population (chi2 test or t-test for independent samples).

**p value <0.001 when comparing the same category between MS-MV and the immigrant population (chi2 test or t-test for independent samples).

### Crude comparison of the health status of the MS-MV and the immigrant population

The group that preferred not to report its migration status has a significantly higher rate of ***any disability*** than the immigrant population (7% versus 4%). There is no significant difference in the prevalence of ***any chronic condition or cancer*** in the past year between theMS-MV and the immigrant population. There is also no difference in the prevalence of ***any health problem or accident*** between the two populations under study, but a significant difference in the proportion of people reporting ***any hospitalization or surgery*** in the missing values versus the immigrants, with higher rates in the later population (4% versus 10%). There is no difference in the mean number of ***medical attentions*** received in the past month between the immigrants and the MS-MV, but a significant difference in the mean number of ***emergency attentions*** received in the past month between the immigrants and the MS-MV (mean 1.1 versus 1.4). Stratified analysis also shows that people with any emergency consultation in the MS-MV are significantly younger than those among the immigrant population (over 50% of them are children). When stratifying the health outcomes by sex, men had almost double the rates of self-reported disability in comparison to women, but this was not statistically significant (4% in immigrant men and 8% in the MS-MV men, against 2% in immigrant women and 5% in MS-MV women). However, both immigrant and MS-MV women showed slightly higher rates of any chronic condition or cancer in the last year (4-5% versus 3%) and around 30% higher rates of any health problem or accident than men (p > 0.05 in all cases) (Table 2).

**Table 2:** Health outcomes of the migration status missing values (MS-MV) and the immigrant population (CASEN survey 2006), overall and by age group and sex

**Health outcomes** (**crude and stratified by age groups and sex**)	**Immigrant population 1%total sample**, **n** = **154 431 weighted population** (**1877 real observations**)		**MS**-**MV GROUP 0**.**67**% **total sample**, **n** = **108 599 weighted population** (**1477 real observations**)	
	**% or mean**	**95% CI**	**% or mean**	**95% CI**
Any disability**	3.55	2.49-5.02	7.42	5.28-10.33
By age groups:				
<16 years old	2.18	0.74-6.23	1.93	0.90-4.11
16–65 years old**	2.96	1.85-4.72	9.67	6.16-14.86
>65 years old	12.39	7.04-20.90	26.42	15.66-40.97
By sex:				
Male	4.33	2.57-7.22	8.85	5.90-13.08
Female	2.90	1.84-4.54	5.89	3.76-9.12
Any chronic condition or cancer	3.90	2.68-5.63	4.26	2.84-6.34
By age groups:				
<16 years old**	0.003	0.00-0.28	2.15	0.84-5.42
16–65 years old	2.83	1.84-4.33	3.89	2.31-6.49
>65 years old	22.61	11.94-38.63	19.29	9.75-34.59
By sex:				
Male	3.68	1.94-6.88	3.72	1.98-6.87
Female	4.09	2.60-6.39	4.83	2.95-7.83
Any health problem/ accident	10.80	8.70-13.32	14.12	11.21-17.65
By age groups:				
<16 years old	6.92	3.80-12.28	12.63	8.77-17.86
16–65 years old	10.44	8.25-13.13	13.51	9.62-18.66
>65 years old	21.36	11.00-37.38	27.02	14.77-44.16
By sex:				
Male	9.09	6.41-12.76	12.19	8.80-16.66
Female	12.14	9.32-15.64	16.19	12.20-21.17
Any hospitalisation/ surgery**	10.80	8.70-13.32	4.59	3.26-6.42
By age groups:				
<16 years old	2.76	0.67-10.62	3.46	1.85-6.38
16–65 years old	5.92	4.28-8.15	5.08	3.16-8.09
>65 years old	16.03	6.85-33.13	8.24	3.37-18.75
By sex:				
Male	4.85	2.63-8.77	4.42	2.63-7.32
Female	7.37	5.24-10.27	4.77	3.06-7.36
Number of medical attentions	*X* = 2.24	1.81-2.66	X = 2.67	1.94-3.40
By age groups:				
<16 years old	1.52	1.15-1.89	2.31	1.53-3.08
16–65 years old	2.19	1.69-2.70	3.27	1.53-3.78
>65 years old	3.19	1.81-4.57	2.66	1.53-3.78
By sex:				
Male	2.43	1.66-3.21	2.08	1.02-3.14
Female	2.11	1.62-2.59	3.36	2.37-4.35
Number of emergency attentions**	*X* = 1.13	1.02-1.25	X = 1.40	1.29-1.60
By age groups:				
<16 years old	1.30	0.85-1.74	1.50	1.19-1.81
16–65 years old	1.11	0.98-1.23	1.28	1.05-1.50
>65 years old	1.19	0.76-1.61	1.08	0.88-1.27
By sex:				
Male	1.11	0.98-1.24	1.44	1.13-1.75
Female	1.15	0.98-1.32	1.36	1.14-1.59

*p value <0.05 when comparing the same category between MS-MV and the immigrant population (chi2 test or t-test for independent samples).

**p value <0.001 when comparing the same category between MS-MV and the immigrant population (chi2 test or t-test for independent samples).

### The relationship between health problems and different sets of social determinants: multivariate models comparing the MS-MV and the immigrants

The significant determinants of ***any disability*** in the MS-MV population are age (OR 1.04), sex (female OR 0.39) and educational level (all categories had a higher chance of presenting with this outcome than those at university level, trend p-value < 0.001). A further analysis comparing SES and material factors indicated a confounding effect was found between SES and material living conditions in the MS-MV group with any disability. Material factors lost significance in the presence of SES, in particular education (data not shown). Immigrants in contrast show a significant association between any disability and age only (OR 1.04). The single significant determinant of ***any chronic condition or cancer*** in those that preferred not to report their migration status is age (OR 1.02), whereas immigrants show a significant association with age (OR 1.05), sex (female OR 2.78) and belonging to an ethnic group (OR 0.08) (Table 3).

**Table 3:** Odds ratio (OR) of presenting **any disability and any chronic condition or cancer**, adjusted by different sets of factors separately (CASEN survey 2006)

	**Any disability**				**Any chronic condition or cancer**			
	**International immigrants**		**MS**-**MV**		**International immigrants**		**MS**-**MV**	
	**OR**	**95% CI**	**OR**	**95% CI**	**OR**	**95% CI**	**OR**	**95% CI**
***DEMOGRAPHICS***								
Age	**1**.04*	**1**.**02**-**1**.**06**	**1**.04*	**1**.**02**-**1**.**06**	**1**.05*	**1**.**02**-**1**.**08**	**1**.02*	**1**.**01**-**1**.**04**
Sex (female = 1)	0.56	0.25-1.25	**0**.39*	**0**.**20**-**0**.**75**	**2**.78**	**1**.**26**-**6**.**71**	1.05	0.46-2.36
Marital status:								
Single	1.00	-	1.00	-	1.00	-	1.00	-
Married	0.79	0.29-2.17	**0**.31*	**0**.**14**-**0**.**72**	3.76	0.25-54.76	1.21	0.46-3.13
Divorced	2.57	0.52-8.73	0.84	0.12-1.52	5.20	0.15-17.21	1.15	0.20-6.43
Widow	1.07	0.26-4.39	0.31	0.07-1.21	-	-	1.51	0.35-7.22
Ethnicity: any	1.06	0.17-6.48	0.89	0.30-2.65	**0**.08**	**0**.**008**-**0**.**07**	1.10	0.19-6.20
Zone (Rural = 1)	1.56	0.80-3.04	0.61	0.28-1.30	0.33	0.04-6.28	0.65	0.26-1.57
Area:								
Northern	1.00	-	1.00	-	1.00	-	1.00	-
Central	0.48	0.14-1.64	0.46	0.15-1.39	0.57	0.21-1.52	1.17	0.31-4.38
Southern	0.89	0.27-2.91	1.22	0.36-4.07	0.93	0.21-1.52	2.55	0.69-9.35
*GOF test*^*α*^	-	<*0*.*001*	-	<*0*.*001*	-	<*0*.*001*	-	<*0*.*001*
***SES*** (***adjusted by age***, ***sex and urban***/***rural***)								
Educational level:								
No education	1.94	0.41-9.12	7.23*	1.05-16.20	**0**.03**	**0**.**001**-**0**.**89**	2.35	0.21-25.42
Primary School	1.95	0.70-5.40	9.65**	1.90-80.41	0.10	0.05-1.90	7.92	0.96-89.94
High School ^b^	1.05	0.37-2.91	4.81*	1.16-38.52	0.78	0.23-2.62	2.04	0.21-19.65
Technical level	0.07	0.01-0.48	6.60**	1.93-72.13	0.48	0.08-2.85	1.37	0.13-14.03
University level	1.00	-	1.00	-	1.00	-	1.00	-
Household income per capita (USD)	0.68	0.18-2.51	2.23	0.50-9.90	0.84	0.12-1.52	0.84	0.18-3.84
Current worker	4.31	0.43-9.63	0.09	0.002-1.98	**0**.56*	**0**.**09**-**0**.**98**	1.44	0.25-8.21
*GOF test*^*α β*^	-	<*0*.*001*	-	<*0*.*001*	-	<*0*.*001*	-	<*0*.*001*
***MATERIAL HOUSEHOLD CONDITIONS*** (***adjusted by age***, ***sex and urban***/***rural***)								
Quality Household:								
Acceptable	1.00	-	1.00	-	1.00	-	1.00	-
Sub-standard	0.90	0.44-1.81	0.10	0.01-6.75	0.78	0.15-2.01	11.27	0.21-38.38
Unfit	4.37	0.86-22.01	0.30	0.01-8.40	0.63	0.06-6.01	-	-
Sanitary Index (adequate = 1)	0.82	0.37-1.81	1.85	0.02-11.35	3.17	0.07-12.66	0.18	0.06-5.71
Overcrowding	0.58	0.26-1.30	0.03	0.001-8.27	0.55	0.02-12.54	5.29	0.60-14.12
HAI	0.94	0.87-1.07	2.24	0.30-5.85	0.76	0.51-1.12	0.31	0.01-8.07
*GOF test*^*α β*^	-	<*0*.*001*	-	<*0*.*001*	-	<*0*.*001*	-	<*0*.*001*

*p value <0.05.

**p value < 0.001.

^*α*^ A p value <0.05 indicates **poor** goodness of fit of the model (logistic regression).

The only social determinant of ***any health problem or accident*** among immigrants is being employed (OR 0.13) and no factors included in the MS-MV analysis showed a significant association with this health outcome. With respect to ***any hospitalization or surgery***, the MS-MV group shows a significant association with being married and widowed (OR 3.68 and 7.88, respectively, Wald test p-value <0.01) and living in the central area of Chile (OR 0.32, Wald test p-value >0.05), while immigrants report a significant association with residence in the Southern area (OR 3.36, Wald test p-value >0.05) (Table 4).

**Table 4:** Odds ratio (OR) or incidence rate ratio (IRR) of presenting **any health problem or accident and any hospitalization or surgery**, adjusted by different sets of factors separately (CASEN survey 2006)

	**Any health problem or accident**				**Any hospitalization or surgery**			
	**International immigrants**		**MS**-**MV**		**International immigrants**		**MS**-**MV**	
	**OR**	**95% CI**	**OR**	**95% CI**	**OR**	**95% CI**	**OR**	**95% CI**
***DEMOGRAPHICS***								
Age	1.02	0.96-1.06	1.002	0.98-1.02	1.00	0.97-1.03	0.98	0.96-1.01
Sex (female = 1)	2.10	0.84-5.22	1.29	0.82-2.91	1.63	0.77-3.41	1.06	0.49-2.27
Marital status:								
Single	1.00	-	1.00	-	1.00	-	1.00	-
Married	2.05	0.82-5.13	1.79	0.85-3.74	1.40	0.54-3.63	**3**.68**	**1**.**22**-**11**.**10**
Divorced	3.84	0.86-17.00	2.96	0.76-11.85	0.16	0.02-1.02	1.57	0.14-6.97
Widow	0.61	0.04-8.52	2.55	0.57-12.82	2.49	0.25-24.76	**7**.88**	**1**.**16**-**15**.**33**
Ethnicity: any	0.60	0.06-5.59	0.54	0.18-1.66	1.11	0.24-5.05	0.35	0.07-1.63
Zone (Rural = 1)	1.96	0.42-9.08	1.09	0.69-1.71	1.23	0.60-2.51	0.64	0.29-1.41
Area:								
Northern	1.00	-	1.00	-	1.00	-	1.00	-
Central	1.35	0.44-4.18	0.98	0.63-2.21	2.69	0.81-8.93	**0**.32**	**0**.**13**-**0**.**78**
Southern	0.44	0.06-2.99	1.33	0.57-3.11	**3**.36*	**1**.**02**-**11**.**05**	0.98	0.40-2.41
*GOF test*^*α*^	-	<*0*.*001*	-	<*0*.*001*	-	<*0*.*001*	-	<*0*.*001*
***SES*** (***adjusted by age***, ***sex and urban***/***rural***)								
Educational level:								
No education	0.10	0.002-4.73	3.86	0.93-15.90	0.31	0.06-1.49	2.66	0.60-11.79
Primary School	0.78	0.21-2.80	1.30	0.35-4.94	0.59	0.22-1.57	1.13	0.24-5.20
High School ^b^	1.006	0.33-2.98	2.11	0.55-8.11	0.82	0.35-1.90	2.10	0.48-9.19
Technical level	0.50	0.10-2.45	0.42	0.08-2.15	1.29	0.50-3.33	-	-
University level	1.00	-	1.00	-	1.00	-	1.00	-
Household income per capita (USD)	0.95	0.13-6.76	1.22	0.58-2.55	1.01	0.99-1.08	0.99	0.98-1.01
Current worker	**0**.13**	**0**.**03**-**0**.**52**	0.69	0.07-6.40	-	<*0*.*001*		
*GOF test*^*α β*^	-	<*0*.*001*	-	<*0*.*001*	-	<*0*.*001*	-	<*0*.*001*
***MATERIAL HOUSEHOLD CONDITIONS*** (***adjusted by age***, ***sex and urban***/***rural***)								
Quality Household:								
Acceptable	1.00	-	1.00	-	1.00	-	1.00	-
Sub-standard	1.98	0.89-4.40	0.10	0.09-1.73	0.75	0.39-1.43	1.12	0.49-2.56
Unfit	6.07	0.50-73.08	1.94	0.001-3.90	0.46	0.07-2.70	-	-
Sanitary Index (adequate = 1)	4.18	0.89-19.63	0.41	0.49-3.96	0.98	0.54-1.77	1.04	0.45-2.41
Overcrowding	0.97	0.37-2.50	0.01	0.004-4.53	0.72	0.37-1.38	1.11	0.54-2.27
HAI	1.09	0.95-1.26	7.17	0.07-12.22	1.02	0.98-1.13	1.05	0.91-1.20
*GOF test*^*α β*^	-	<*0*.*001*	-	<*0*.*001*	-	<*0*.*001*	-	<*0*.*001*

*p value <0.05.

**p value < 0.001.

^*α*^ A p value <0.05 indicates **poor** goodness of fit of the model (logistic regression).

The factors associated with the number of ***medical attentions*** received in the past three months in the MS-MV are sex (female PRR 1.76) and educational level (all categories with a higher risk ratio than the university level, no gradient, Wald test p-value <0.01). In contrast, among immigrants age (PRR 1.01, continuous variable) and living in a rural setting (PRR 0.67) affect the number of medical attentions received in the past three months. There is a partial confounding effect between age and medical attentions, as the magnitude of this association is weakened by 15% in the presence of education level. Both covariates nevertheless remain independently associated with this outcome.

Employment status (PRR 0.29) is the only variable significantly associated with the number of ***emergency consultation***s received in the past three months in the MS-MV group (PRR 0.29) and no factor was significantly associated with this health problem in the immigrant population (Table 5).

**Table 5:** Prevalence rate ratio (PRR) of the number of **medical and emergency attentions received** in the past month, adjusted by different sets of SDH separately (CASEN survey 2006)

	**Number of medical attentions**				**Number of emergency attentions**			
	**International immigrants**		**MS**-**MV**		**International immigrants**		**MS**-**MV**	
	**PRR**	**95% CI**	**PRR**	**95% CI**	**PRR**	**95% CI**	**PRR**	**95% CI**
***DEMOGRAPHICS***								
Age	**1**.01*	**1**.**005**-**1**.**02**	1.001	0.98-1.01	1.004	0.98-1.02	0.99	0.98-1.002
Sex (female = 1)	0.98	0.45-2.11	**1**.76*	**1**.**14**-**2**.**72**	1.51	0.84-2.71	1.02	0.79-1.32
Marital status:								
Single	1.00	-	1.00	-	1.00	-	1.00	-
Married	1.14	0.84-1.55	1.82	0.97-3.39	1.30	0.46-3.67	1.30	0.77-2.17
Divorced	0.89	0.54-1.49	1.55	0.77-3.44	0.77	0.18-3.20	0.90	0.56-1.45
Widow	1.007	0.57-1.77	0.62	0.26-1.48	1.96	0.30-12.70	1.41	0.64-3.11
Ethnicity: any	0.86	0.59-1.25	0.85	0.44-1.62	0.72	0.25-2.06	0.93	0.41-2.12
Zone (Rural = 1)	**0**.67*	**0**.**49**-**0**.**91**	0.95	0.61-1.46	1.09	0.54-2.21	1.31	0.74-2.34
Area:								
Northern	1.00	-	1.00	-	1.00	-	1.00	-
Central	1.93	0.92-4.04	0.90	0.48-1.68	0.71	0.21-2.33	1.17	0.91-1.50
Southern	1.31	0.68-2.52	0.68	0.36-1.27	0.66	0.18-2.35	1.33	0.90-1.95
*GOF test*^*α β*^	-	<*0*.*001*	-	<*0*.*001*	-	<*0*.*001*	-	<*0*.*001*
***SES*** (***adjusted by age***, ***sex and urban***/***rural***)								
Educational level:								
No education	0.39	0.02-7.22	**3**.54**	**2**.**24**-**5**.**61**	3.08	0.63-14.97	1.34	0.90-1.98
Primary School	1.57	0.17-14.55	**1**.57*	**1**.**09**-**2**.**26**	2.05	0.51-8.27	0.99	0.65-1.49
High School ^b^	0.44	0.12-1.62	**4**.33**	**2**.**14**-**8**.**73**	3.35	0.89-12.49	0.82	0.55-1.23
Technical level	0.36	0.05-2.39	2.09	0.99-4.41	4.74	0.95-23.49	0.89	0.43-1.83
University level	1.00	-	1.00	-	1.00	-	1.00	-
Household income per capita (USD)	0.99	0.99-1.01	1.63	0.92-2.87	1.001	0.99-1.003	1.07	0.72-1.60
Current worker	0.80	0.42-1.62	0.81	0.22-2.21	0.65	0.39-1.09	**0**.29**	**0**.**34**-**0**.**59**
*GOF test*^*α β*^	-	<*0*.*001*	-	<*0*.*001*	-	<*0*.*001*	-	<*0*.*001*
***MATERIAL HOUSEHOLD CONDITIONS*** (***adjusted by age***, ***sex and urban***/***rural***)								
Quality Household:								
Acceptable	1.00	-	1.00	-	1.00	-	1.00	-
Sub-standard	0.85	0.66-1.10	0.57	0.001-26.86	1.009	0.84-1.20	0.96	0.06-13.21
Unfit	0.61	0.37-1.01	0.02	0.001-5.21	0.87	0.73-1.04	0.40	0.02-42.15
Sanitary Index (adequate = 1)	1.32	0.54-3.21	5.12	0.01-8.27	0.93	0.64-1.36	1.46	0.04-46.28
Overcrowding	0.86	0.36-2.00	0.02	0.001-32.12	1.56	0.90-2.73	0.86	0.02-36.56
HAI	0.33	0.02-4.25	3.21	0.01-12.45	0.55	0.90-2.73	3.92	0.01-9.62
*GOF test*^*α β*^	-	<*0*.*001*	-	<*0*.*001*	-	<*0*.*001*	-	<*0*.*001*

*p value <0.05.

**p value < 0.001.

^*α*^ A p value <0.05 indicates **adequate** goodness of fit of the model (negative binomial regression over Poisson regression, alpha test).

^*β*^ A p value <0.05 indicates **adequate** goodness of fit of the model (zero-inflated negative binomial regression over non zero-inflated regression, Vuong test).

Goodness of fit of the logistic models were poor in most cases (except for partial models of migration-related factors), but all negative binomial models showed a better fit than a regular Poisson regression (alpha test) and all zero-inflated models showed a better fit than non zero-inflated regressions (Vuong test).

## Discussion

### Summary of main findings

**Figure 1:**
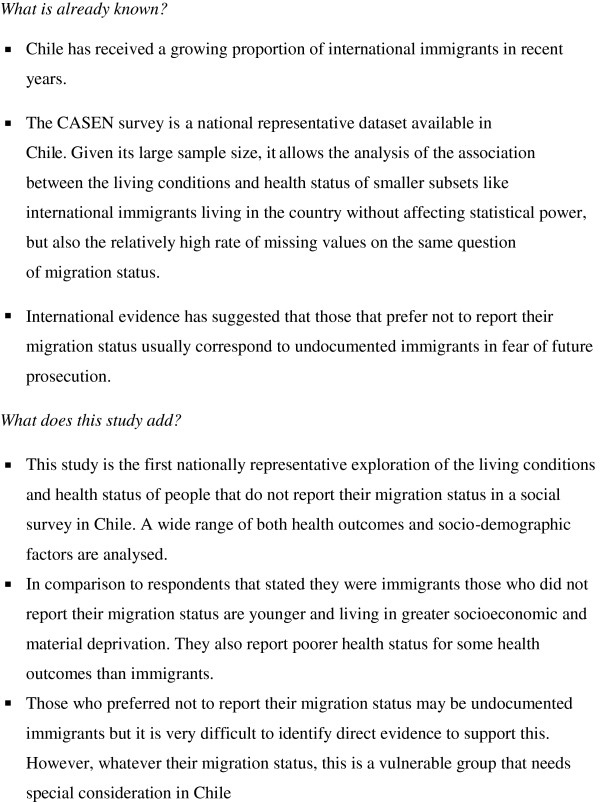
Summary of contribution to evidence from this study.

A summary of key findings and the contribution of this paper to current evidence on migration and health appear in Figure 1. This study is the first national-representative exploration of the living conditions and health status of people that prefer not to report their migration status in a social survey in Chile. A wide range of both health outcomes and socio-demographic factors were analysed. People that do not report their migration status are more likely to be children. Also, the MS-MV tend to live in higher socioeconomic and material deprivation than those that respond that they are immigrants, and their health status is poorer than them for most health outcomes included in this study. When stratifying the health outcomes by sex, men report almost double the levels of disability of women, but this was not statistically significant. However, both immigrant and MS-MV women showed slightly higher rates of any chronic condition or cancer and around 30% higher rates of any health problem or accident in the last month than men. Regression models indicated that age, sex, SES and material factors significantly affected the chance of poor health and their associations with health varied between MS-MV and self-reported immigrants.

### Are the missing values cases undocumented immigrants?

As described above, the CASEN survey does not contain any direct evidence regarding the legal status of respondents that choose not to report their migration status. However, the markedly higher rates of non-response to the CASEN migration status question in comparison to all other questions in the survey suggest that there were distinctive reasons for non-response to this question and that it may have represented a sensitive subject for some respondents. In addition the characteristics of the MS-MV identified in this analysis, including young age, low socio-economic status and poor health are compatible with previous research describing undocumented immigrants. International evidence has suggested that those that do not report their migration status may be commonly undocumented immigrants [11,28,29] and under-estimation of immigrants in national representative surveys has been frequently reported in the past [1,3,30,31,48]. It is also notable that when the immigrants and the missing values are added together, they account for 1.63% of the total Chilean population, which is very similar to the prevalence of immigrants in the Chilean population recently reported by the Chilean government (1.6-1.8%) [15,35]. These national statistics are considered to be accurate and robust, and come from the Migration Department in Chile based upon a detailed analysis of people moving in and out of the country every year.

While we cannot confirm the legal status of the MS-MV in this study, the findings indicating their socioeconomic and health vulnerability may have some implications for understanding immigrant health in Chile. These results suggest that social and health policies in Chile designed to protect immigrants may be inadequate. Most of these policies are available to documented immigrants only (e.g. access to the public healthcare system and all related programmes), with a few exceptions like antenatal care, emergency care, and infant care (Resolution No. 1914 of March 13, 2008 and REGULAR 14 Number 3 229, of June 11th, 2008; Consulado General del Perú en Santiago de Chile, 2009) [49]. These measures, implemented in the last 5–10 years, are important for health but they might not be available to all immigrants on their arrival to Chile. Further studies need to address limitations of entitlement to these policies. An extension of our analysis could consider healthcare entitlement by immigrants and the MS-MV group and how this varies by SES, gender, sex and other possible determinants of access to healthcare in a middle-income country with a mixed public and private healthcare system like Chile. Findings from this study, however, indicate that there is an opportunity for the Chilean government and non-governmental organizations to become the bridge between international immigrants and the social and healthcare systems in Chile and the Latin American region, particularly among the women, the socio-economically disadvantaged, the unemployed, and those living with a large number of children.

### Undocumented immigrants: a hard to reach population?

Limited international data available on undocumented immigrants suggest they are extremely vulnerable to lower self-reported health, accidents, injuries, and psychosocial distress resulting from poor working conditions [1,50-54] and marginal living conditions, associated with poverty, social exclusion, and discrimination [55]. Reasons for this are multiple and complex, including socioeconomic deprivation, social isolation, experiences of stigma and discrimination, language limitations, higher rates of isolated ethnic groups, psychological stress in the migration process and arrival in the host country, changes in legal status, fear of prosecution and deportation.

Legal status (documented versus undocumented) is a particular sensitive subject, potentially leading to self-selection out of survey participation, skewing results and limiting the study of the impact of legal status on the relationship between migration and health [1,56,57]. In Chile, legal status among immigrants can be granted through three main mechanisms, temporary residency usually for a year, permanent residency or tourism, but may be lost after a year or when an employment contract expires [49]. There is a well-documented relationship between legal status and contractual status among immigrants in the international literature [58,59], which is also found for some health problems in this study, but surely requires further understanding in Chile.

### Methodological limitations

Five main limitations of this study’s methodology can be recognised. First, this study comes from secondary analysis of a large survey and therefore key variables relevant to migration status and legal status were not available for analysis and interpretation. Questions on legal status, reasons for not wanting to declare migration status, perceptions of stigma and discrimination, and others, could not be directly assessed in this study and need further consideration in Chile. Second, the data used in our study belonged to a cross-sectional design and we cannot determine whether migration is a cause of poor SES or poor health [60]. However, the causal relationship between migration, SES and health has been widely discussed in past decades and findings from this study help us raise hypotheses on the importance of SES in the health status on immigrants and the MS-MV for further debate and research in the region and elsewhere [61-63]. Third, there is an evident risk of self-report bias in this study, not only of migration status, but also SES and ill health. However, although some limitations of these measures have been recognized, they are considered fairly robust measures and are widely used in health research [64]. Fourth, great heterogeneity in the socio-demographics of both the MS-MV and the immigrants was observed, as seen through wide confidence intervals in regression models. This is not due to lack of statistical power but results from the great variation within the groups under study. This is not uncommon for immigrant groups that encompass a range of subsets of families with different backgrounds. Such heterogeneity did not allow us to observe all covariates that may be significantly associated with migration status. Future more focused quantitative studies on immigrants to Chile perhaps could better capture this heterogeneity and would be of great help in understanding the living conditions and health of immigrants to Chile. Fifth, many of the predictor variables are likely to be highly associated with household income. This has been dealt with by building models using only a few predictors at a time. However, risk of endogeneity or correlation between covariates in regression models remains a significant issue and is a further limitation of our analysis.

### Research and policy implications

Self-reported migration status is a particularly sensitive measure and misrepresentation of immigrants through population-based surveys is well established [65,66]. Efforts have been made to improve the representation of the international migrant population in research, but current research methodologies are being challenged by the complexity involved when studying hard to reach populations, such as migrants without legal status. This study confirms the need for concrete national research strategies to overcome these limitations in Chile. Latin America and the world as a whole face a great challenge developing culturally sensitive approaches to these groups.

International evidence on specific sampling strategies for hard to reach populations; qualitative research on specific vulnerable immigrant groups, and tools for consistent data collection in nation-wide surveys could be considered to address these issues [50,67-69]. Regarding the CASEN survey in particular, reasons for not wanting to report migration status could be collected, along with the addition of questions on migrants’ legal status, contractual status and reasons for immigration to Chile. Interviewing skills for field teams could be strengthened by putting greater focus upon the reassurance of anonymity of the information collected in the survey and the significant future policy implications of accurate data collected among immigrants in Chile, to protect their health and living conditions. Further survey questions on working conditions, perceived stigma and discrimination, and self-reported general health would further improve understanding of immigrant health. Studies such as this one need to be complemented with more focused surveys of immigrants in Chile, and also more ethnographic qualitative analysis. This could provide a better representation of the true living conditions and health of immigrants in Chile, both documented and undocumented.

In relation to policy and public health implications of the key findings from this study, they suggest the existence of a great complexity and significant vulnerability in the health, socio-demographic characteristics and living conditions of those that prefer not to report their migration status in Chile. There is no simple story to tell in terms of their health status and factors associated with their health conditions. This analysis however suggests that these people may represent a vulnerable, hard to reach group of immigrants and tailored interventions and programmes may be needed to identify these groups and to improve the health.

## Conclusion

The under-representation of undocumented immigrants in surveys is a significant problem in migration research. This study explored the living conditions and health of those who preferred not to report their migrations status (MS-MV) in the Chilean CASEN-2006 survey and compared them to international immigrants. Results from this study contribute to the current understanding of the health of international immigrants in Chile and the Latin American region. Several hypotheses have been raised to explain the distinctive socio-demographic and health patterns that have been observed among immigrants and the missing values, which could be assessed with better data in the future. The MS-MV lived in more deprived conditions than immigrants, and reported a higher rate of health problems than immigrants. This study highlights the urgent need to find more effective ways to reach potential undocumented immigrants in fear of prosecution. Better data about this group will support improved public health interventions to protect vulnerable underserved marginalised immigrants in this region and beyond.

## Abbreviations

MS-MV: Missing status missing values those that preferred not to report their migration status in the 2006 CASEN survey; SES: Socioeconomic status; HAI: Household asset index estimated through principal component analysis (PCA); GOF: Goodness of fit.

## Competing interests

The authors declare that they have no competing interests.

## Authors' contributions

The three authors made substantial contributions to conception and design, analysis and interpretation of data, drafting of the manuscript, and final approval of the version to be published. BC developed the study design, conducted the analysis and drafted the manuscript. KP participated in the design, analysis, interpretation and drafting of this paper. HT participated in the design, analysis, interpretation and drafting of this paper. All authors read and approved the final manuscript.

## Pre-publication history

The pre-publication history for this paper can be accessed here:

http://www.biomedcentral.com/1471-2458/12/1013/prepub
